# Amino Acid Analyses of Plant Foods Used in the Dietary Management of Inherited Amino Acid Disorders

**DOI:** 10.3390/nu15102387

**Published:** 2023-05-19

**Authors:** Suzanne Ford, Fatma Ilgaz, Sarah Hawker, Barbara Cochrane, Melanie Hill, Charlotte Ellerton, Anita MacDonald

**Affiliations:** 1 National Society for Phenylketonuria (NSPKU), Sheffield S12 9ET, UK; 2 Southmead Hospital North Bristol Trust, Bristol BS10 5NB, UK; 3 Department of Nutrition and Dietetics, Faculty of Health Sciences, Hacettepe University, Ankara 06100, Turkey; fatma.celik@hacettepe.edu.tr; 4 Alta Bioscience, Redditch B98, UK; info@altabioscience.com; 5 NHS Greater Glasgow and Clyde, Royal Hospital for Children, Glasgow G51 4TF, UK; barbara.cochrane@ggc.scot.nhs.uk; 6 Sheffield Teaching Hospitals NHS Foundation Trust, Sheffield S5 7AU, UK; melanie.hill13@nhs.net; 7 University College London Hospitals NHS Foundation Trust, London WC1N 3BG, UK; c.ellerton@nhs.net; 8 Birmingham Children’s Hospital, Steelhouse Lane, Birmingham B4 6NH, UK; anita.macdonald@nhs.net

**Keywords:** inherited metabolic disorders, amino acids, phenylalanine, tyrosine, methionine, leucine, lysine, fruits, vegetables, plant, protein

## Abstract

A low amino acid (AA)/protein diet is the principal treatment for many inherited amino acid disorders (IMDs). Due to their low AA content, plant foods constitute an essential part of diet therapy. However, data on their AA composition are limited, which leads to an estimation of AA intake from protein content rather than an accurate calculation of true AA intake. This study describes the AA content of a total of 73 plant foods (fruits, *n* = 12; vegetables, *n* = 51; and other plant foods, *n* = 10), with the analysis commissioned by the UK National Society for Phenylketonuria (NSPKU) over 15 years. For all fruits and some vegetables (e.g., rocket, watercress and pea shoots), raw samples were used during analysis. All other vegetables were cooked prior to analysis to represent the usual condition of the food at the time of serving. AA analysis was performed with ion exchange chromatography. The median percentage of protein was 2.0% [0.6–5.4%] for the fruits and vegetables analysed (*n* = 56), although higher in vegetables than in fruits. Each of the five reported AAs (leucine, lysine, phenylalanine, tyrosine, and methionine) supplied 1–5% per g of protein content. From the heterogeneous range of plant foods analysed, the AA/protein ratios differed significantly (2–5% in fruits and 1–9% in vegetables). There was a strong correlation between the amounts of each of the five AAs in the plant foods, but only a small, moderate correlation between the protein and AA content. Overall, this study provides data on the AA content of several plant foods, which are suitable for patients treated with a low AA/protein diet, including many novel plant options. However, only a limited range of fruits and vegetables were analysed due to the high costs of analysis. Hence, more extensive studies with an increased number of plant foods prepared by different cooking methods and replicate samples are necessary, particularly to examine the relationship between the protein and AA content in depth.

## 1. Introduction

Inherited metabolic disorders (IMDs) treated with low amino acid diets [amino acidopathies; phenylketonuria (PKU), maple syrup urine disease (MSUD), homocystinuria (HCU), type I, II, and III tyrosinemia (TYR I, II and III), organic acidemias; glutaric aciduria type I (GAI)] are a group of rare genetic disorders caused by enzyme deficits in the metabolic pathway of amino acids (AAs) [1,2,3]. Early diagnosis and life-long treatment are essential to prevent signs and symptoms affecting multi-organ systems, including neurodevelopmental deficits, metabolic encephalopathy, liver dysfunction, thromboembolism, and osteoporosis [1,4,5,6,7,8]. Dietary management is the principal treatment for all these disorders. It involves natural protein/precursor AA restriction, exclusion of high-protein foods (e.g., meat, fish, eggs, and chicken), the addition of a precursor-free protein substitute, and special low-protein foods (e.g., bread, pasta). Although there are some pharmaceutical options that enable dietary relaxation, this is only in a sub-section of patients with PKU [9,10] or homocystinuria [11].

Due to their low protein content (<3–8% in vegetables; <1–1.5% in fruits) [12], fruits and vegetables constitute an essential part of dietary management. They have many benefits. They provide variety; necessary vitamins, minerals, fibre and phytochemicals [13]. Fibre and polyphenols are known to promote a healthy gut microbiota [14,15], and all the nutrients have health-promoting effects, including a reduced risk of cardiovascular disease [16,17], certain types of cancer [17] and cognitive disorders [18]. Given these benefits, the World Health Organization (WHO), recommends a daily intake of at least five portions (i.e., 400 g) of fruits and vegetables daily (excluding potatoes, sweet potatoes, cassava, and other starchy roots) [19]. Many patients with classical forms of amino acid disorders tolerate less than 10 g/day of natural protein, which severely limits the amount of natural food that can be eaten, including fruits and vegetables. Depending on their protein and AA content, some fruits and vegetables can either be given without measurement (i.e., exchange-free food) or they must be calculated/measured as a part of the daily protein/amino acid allowance. Thus, the determination of the protein and AA content of fruits and vegetables is essential. This will help metabolic dietitians perform accurate dietary calculations so they can give appropriate guidance to patients and achieve optimal metabolic control.

Overall, there are around 3000 known fruits and vegetables. However, there is limited data on their AA content. In the UK, the last amino acid dataset was published in 1980 [20], and the number of foods analysed for AA content was only 1200 with a narrow range of analysis for fruits and vegetables. The low interest in performing the AA analysis of foods can be explained by high analysis costs (around GBP 400 per sample for food analysis), lack of AA food labelling requirements, and limited relevance for the general population. Hence, in IMD, the current practice is to commonly estimate the AA content of foods from their protein content. For some AAs, such as phenylalanine (Phe), it is recognised that protein contains around 5% Phe in foods such as animal milk and cereal protein sources. However, this is not consistent for fruits and vegetables, as their protein content contains a lower amount of Phe (3–4%). In a technical report published by Kim et al. [21], the minimum and maximum Phe/protein ratios have been reported as: 20–39 mg Phe per 1 g of protein for fruits and 20–40 mg Phe per 1 g of protein for vegetables except for spinach, peas, seaweed, kale, and sweetcorn, which have a higher ratio. Similar inconsistencies apply to the protein amounts of other AAs, including lysine (Lys), leucine (Leu), valine, isoleucine, and methionine (Met). Their contents vary widely, from 1 to 6% in fruits and vegetables, and even wider in cereals and milk products, from 2 to 11% [1,20]. It is essential that reliable AA information be accessible for plant foods that may be utilised in a low protein/AA diet. New plant food options are regularly introduced, but commonly, there is no or limited knowledge about their AA content. The availability of this information would potentially lead to their inclusion, thereby improving dietary diversity, nutrient intake, and satiety for patients with IMD.

The National Society for Phenylketonuria (NSPKU) has a long history of analysing fruit and vegetables for their Phe content [22]. In recent years, the NSPKU has extended the AA analysis to include a wider range of AA in addition to Phe, in order to benefit non-PKU AA disorders.

In this study, we (1) present the AA analysis of different plant foods analysed over the last 15 years, and (2) examine if there is a correlation between the protein and AA content of plant foods in order to facilitate an estimation of the AA content of plant foods from their protein content.

## 2. Materials and Methods

### 2.1. Selection, Purchase and Preparation of Foods

Four experienced specialist metabolic dietitians, members of the Medical Advisory Panel of the NSPKU, selected the plant foods to be analysed. The food samples were prepared by SF. The following plant foods were prioritised:

1. Any fruits and vegetables with limited information about their AA content, e.g., banana blossom, rainbow and ruby chard, eddoes, breadfruit, callaloo, ackee, and lotus roots.
2. Manufactured fruits and vegetables prepared by different methods that may alter AA content, e.g., vegetable or fruit crisps; sundried tomatoes.
3. Plant flours and other unusual or miscellaneous manufactured plant-based products.

Most of the food products were obtained from UK supermarkets (e.g., Sainsbury’s, Tesco, and Waitrose) to ensure that the food was representative of foods consumed by the UK IMD community. Due to limited financial resources, only a single AA analysis of foods was conducted, but if there was concern that a single analysis may not yield reliable results, multiple analysis was conducted e.g., avocado, sundried tomatoes, and sweet potato chips. For these foods, food samples were purchased from different supermarkets. Care was taken to purchase fruits and vegetables that were fresh, mature and free from bruises or damage. Some fruits or vegetables were frozen (lotus roots) or tinned (jack fruit, breadfruit, and callaloo), as this was the usual available source.

For fruits, only raw samples were used during the analysis. Many of the vegetables were cooked (boiled or steamed until tender) prior to analysis to represent the usual condition of the food at the time of serving. Some vegetables were raw when analysed, e.g., rocket, watercress and pea shoots.

Prior to amino acid analysis, and under laboratory conditions, a 10 g sample of each solid food (raw, cooked or tinned) was homogenised by freezing with liquid nitrogen. It was then blended in a food processor to ensure a representative sample was taken for analysis. For liquid samples, 1 mL of sample was used. All samples were placed in sealed bags and stored at −20 °C for 24 h prior to analysis.

### 2.2. Chemicals and Reagents

All chemicals and reagents used in the AA analysis, including hydrochloric acid (HCl), loading buffer (0.1 M HCl), ninhydrin and sodium citrate buffers, were prepared by Alta Bioscience (Redditch, UK). Constant boiling HCl (5.8 M HCl) was made up with a mixture of 800 mL of concentrated HCl and 800 mL of distilled water. The distillate was then collected at 107–109 °C. Loading buffer was prepared from the HCl stock (approximately 5.8 M) by mixing 17.2 mL of HCl with 1 g of phenol and 1 mL of 2,2-thio-diethanol. The mixture was made up to 1000 mL with Elga water.

### 2.3. Analytical Methodology

The amino acid analysis, first developed by Moore and colleagues in the early 1950s [23,24], was comprised of three main steps. Step 1 involved the hydrolysis of individual AAs from the protein backbone (i.e., release of AAs from the food matrix); step 2, separation of individual AAs using a chromatographic procedure; and step 3, detection and quantification of the separated AAs using calibration standards [25,26].

In this study, the conventional acid hydrolysis method (liquid phase) was used for protein/peptide hydrolysis preceding AA analysis. The samples (100 ± 10 mg) were hydrolysed at 110 °C for 24 h with 6M HCl (Ph.Eur.2.2.56-Method 1) [27].

Samples were then analysed on a Waters 2695 pump/injector system. The AAs were separated by ion exchange chromatography on a strong cation exchange resin using sodium citrate buffer gradients of increasing pH. The ninhydrin reagent was pumped using a Waters 1515 isocratic pump. The ninhydrin reaction occurs in a heated coil at 125 °C situated in a modified column heater. The derivatized AAs are detected in a Waters 2487 variable wavelength UV/VIS detector. Data handling was performed with a Lab Systems “Atlas” integration package. Only one replicate per sample was performed. This method enables the detection of 0.01 nmoles/mg of individual amino acids.

All analyses were performed at AltaBioscience Laboratory (Redditch, UK), accredited to 17025:2017 for AA analysis. The protocol is described in the European Pharmacopoeia 2.2.56 [27].

### 2.4. Data Analysis

The Phe, Leu, Met, Tyr, and Lys content of 73 plant foods was evaluated. Data on the protein content of foods (g/100 g of food) were obtained from different sources, including the UK [28] and US food composition databases [29], nutritional information on the packaging stated by manufacturers and from published literature [22,30].

A pooled analysis (mean ± SD, median and range) was conducted using data on the protein and AA content of a total of 56 fruits and vegetables. The percentage (%) and the amount (mg) of AAs per gram of protein were calculated. The correlation between protein and AA contents of fruits and vegetables was tested by Spearman’s correlation coefficient. Some fruits and vegetables were not included in the pooled analysis. Fruit crisps (*n* = 4) were excluded as their nutritional value significantly deviated from that of fresh fruits due to the loss of water content during manufacturing. Sweet potato fries (*n* = 3), which were lightly coated with flour (e.g., rice, amaranth, corn flour), were also excluded, as their extra ingredients increased protein content and altered the amino acid profile.

## 3. Results

The amino acid composition of different plant sources (*n* = 73), including fruits (*n* = 12), vegetables (*n* = 51), and plant-based foods (*n* = 10), was commissioned by the NSPKU over 15 years (Table 1). Eight fruits and fourteen vegetables were raw; thirty-two vegetables were tinned or cooked. The remaining foods were fruit crisps (*n* = 4), sundried/slow-roasted tomatoes (*n* = 7), and various plant foods in different dry states, e.g., plant powder and plant flours.

### 3.1. Amino Acid Analysis of Fruits

Most fruits had a low Phe content of ≤50 mg/100 g. Exceptions were melon crisps, pineapple crisps and passion fruit, which had a Phe content that exceeded 100 mg/100 g. They also had a high Leu (>100 mg/100 g) and Met (>25 mg/100; equivalent) content (Table 1).

Multiple samples of avocado (*n* = 4) and apple crisps (*n* = 2) were analysed. The AA composition of the different samples of each food was similar. The protein content of apple crisps was variable, but this data was obtained from the food nutritional analysis on product packaging (Table 1).

With the exclusion of fruit crisps (*n* = 4), the protein content of fruits ranged from 0.8–2.6 g/100 g (Table 2).

The percentage of protein provided by individual AAs ranged from 1–5% per 1 g of protein, with the highest contribution from Leu (5%), Lys (3%), and Phe (3%), followed by Tyr (2%) and Met (1%) (Table 3). The minimum and maximum AA/protein ratios for fruits varied from 12 to 45 mg/g of protein (<50 mg/g of protein) (Table 3).

We evaluated the correlation between the protein and AA content of fruits, although the number of fruits analysed for AA composition was limited (*n* = 8). The results indicated a moderate to high correlation between the amount of protein and AAs in fruits (*r =* 0.74 for Phe; *r* = 0.80 for Leu; *r* = 0.63 for Met; *r* = 0.57 for Tyr; and *r* = 0.80 for Lys). There was also a moderate-to-strong positive association between the five AAs in fruits (*r* = 0.58 to 0.98; (Supplementary Table S1).

Overall, fruits had a lower AA content compared to vegetables and some plant flours. Most fruits tested had a low AA content, except for passion fruit and fruit crisps. There was no specific AA/protein ratio (12 to 45 mg/1 g protein in fruits) observed, so it was not feasible to estimate individual amino acid concentrations from their protein content.

### 3.2. Amino Acid Analysis of Vegetables

The vegetables analysed had a low AA content, but in general, their AA content per 100 g was higher than in fruits (Table 1 and Table 2). Only 18% of the vegetables (*n* = 9) analysed had a Phe content of <50 mg/100 g. It was greater than 75 mg/100 g in more than half of the vegetables analysed (*n* = 28). For MSUD, 30 of 51 (59%) vegetables had a Leu content > 100 mg/100 g, and for HCU, 26 of 51 vegetables (51%) had a Met content of >25 mg/100 g.

Multiple samples were analysed for sundried tomatoes (*n* = 5), slow-roasted tomatoes (*n* = 2), and sweet potato fries (*n* = 3). There were significant differences between the protein (data obtained from manufacturers) and AA composition of different brands. For some of the AAs, the difference between the products with the lowest and highest AA content was approximately 2-fold (e.g., Phe content of sundried tomatoes from Baresa vs. Marks & Spencer) (Table 1). Manufactured sweet potato fries (frozen, oven-baked), which were all coated with plant flour, had a higher protein and AA content (≈3-fold) compared to home-cooked sweet potatoes.

Excluding flour-coated sweet potato fries (*n* = 3), the range of protein in vegetables was 0.6–5.4/100 g) (Table 2).

The percentage of protein provided by individual AAs ranged from 1–5% per 1 g of protein. The highest contribution was from Leu (5%), Lys (5%), and Phe (4%), followed by Tyr (2%) and Met (1%) (Table 3). The minimum and maximum AA/protein ratios for vegetables ranged between 11 and 54 mg/1 g of protein (Table 3).

A correlation analysis was calculated for vegetables (*n* = 48). There was a moderate positive association between the amount of protein and AAs (*r* = 0.61 for Phe; *r* = 0.57 for Leu; *r* = 0.52 for Met; *r* = 0.55 for Tyr; and *r* = 0.61 for Lys) and a moderate-to-strong positive association between all five AAs reported in vegetables (Supplementary Table S1).

There are large differences between the protein and AA composition of vegetables and so no specific AA/protein ratio was observed.

### 3.3. Other Plant Foods

Ten plant-based foods were analysed for their AA composition (Table 1). Their protein content ranged from 0–8.3 g/100 g, but their AA content was variable depending on the type of plant. Konnyaku, mung bean vermicelli, and potato flour had the lowest protein and amino acid content. Acai berry powder and green banana flour had the highest. There were discrepancies between the protein and AA composition of similar but different branded products, e.g., rice noodles.

## 4. Discussion

Since the early 1990s, the NSPKU has commissioned the AA analysis of many plant foods in order to safely broaden the range of foods given to patients with PKU. They first reported the Phe content of 172 foods in 2006 [22]. In our paper, we report the analysis of five key AAs in seventy-three plant foods that may be useful in low-AA diets recommended for MSUD, HCU, TYR I, II and III, and GAI. The median percentage of protein was 2.0% [0.6–5.4%] for the fruits and vegetables analysed (*n =* 56), although higher in vegetables than in fruits. Each of the five AAs is supplied at 1–5% per g of protein content.

In human nutrition, the AA composition of foods is not considered a primary concern. A typical balanced diet based on animal and plant protein sources provides a satisfactory or even excessive intake of protein and essential AAs. However, patients diagnosed with inherited AA disorders are commonly treated with a lifelong low-AA/low-protein diet. In this group of patients, both protein adequacy and the measured intake of specific AAs are important to prevent the accumulation of toxic compounds caused by the defect in the metabolic pathway and to maintain blood levels of affected AAs within a target therapeutic range. The availability of reliable data on the AA content of novel plant sources facilitates improved accuracy of dietary calculations and, therefore, their careful inclusion in the dietary management of these rare conditions. Knowledge about the AA content of foods, such as dudhi, eddoes, ackee, banana blossom, breadfruit, callaloo, and lotus roots expands the range of foods that can be offered and is important for patients from a wide range of cultural and ethnic backgrounds. It is well established that the incidence of IMDs may be 10 times higher in groups with high consanguinity rates [31,32]. They are particularly prevalent in people from the Middle East, South Asia, Turkey and North Africa [33], so it is important to include staple foods eaten globally.

In practice, the most common method of calculating the AA intake is to estimate the AA content from food protein (e.g., assuming 1-g protein is equivalent to 50 mg of Phe in PKU or 0.5-g protein is equivalent to 50 mg of Leu in MSUD). Unfortunately, this is inaccurate as the AA contribution to the total protein content is inconsistent between plants and animal foods and even between similar plants [21,34]. For example, we found variations in the Phe content between Ruby and Rainbow chard and sugar snap peas and mange tout.

Proteins, though commonly representing less than 1% of the fresh weight of fruit and vegetable tissues, are structural constituents that are the major solid components of the cytoplasm of living cells [35]. Legumes are the richest in protein, containing around 8% following preparation [28]. Some leafy vegetables and sweet corn contain over 4% protein, but in most other products, the amount is below 3%. The protein content of fruits is usually particularly low, seldom rising above 1.5% and in many cases falling considerably below 1%. Enzyme systems in fruits always contain a protein fraction [12]. The protein content of fresh fruits or vegetables is calculated by multiplying the total nitrogen content by a factor of 6.25 [12]. This calculation uses the fact that protein contains approximately 16% nitrogen, and the assumption that all nitrogen present is protein. However, it does not consider that appreciable amounts of simple nitrogenous substances can be present in an uncombined form. These include free AAs, chlorophylls, polyamines or alkaloids. Free AAs and related amines such as asparagine and glutamine, normally those that are also present in the proteins of the tissue, appear to make up the bulk of non-protein nitrogen [12,35]. The actual proportion of non-protein nitrogen is greatly variable, and there is little accurate quantitative data about this. It is estimated that in potatoes, 50 to 60% of the nitrogen occurs in the form of simple soluble constituents, while in apples, estimates range from 10 to 70% [36]. In our report, we did not analyse the protein content of plant foods but documented this from different sources, including food databases, manufacturers data or published literature.

The mean percentage contribution per g of protein from the five reported AAs for the fruits and vegetables analysed was Leu (5%), Lys (3–5%), and Phe (3–4%), followed by Tyr (2%) and Met (1%). This is similar to previous reports [18], although Daly et al. [37] reported for fruits and vegetables that the Tyr content mainly varied from 1–4% of protein content. From the heterogeneous range of plant foods analysed, the AA/protein ratios differed significantly; e.g., for Phe, AA/protein ratios (%AA per g of protein) was from 2–5% in fruits, and from 1–9% in vegetables. There was a strong correlation between the amounts of each of the five AAs in the plant foods, but only a small, moderate correlation between the protein and AA content. We have only reported the amino acid content of a limited range of fruits and vegetables, so more data from a wider number of plant foods is necessary to examine the relationship between the protein and AA content in depth. Recently, Daly et al. [37] showed a clear and close correlation between Tyr and Phe content in fruit and vegetables. The correlation between Tyr and protein was lower but still apparent [37].

There are many factors that should be considered when interpreting the protein and AA composition of plant foods. Firstly, the AA content of fruits and vegetables may be analysed in different forms, such as raw vs. cooked, fresh vs. processed (e.g., canned, frozen, dried), or coated with flour or milk. This influences the protein and hence the AA content of foods. For example, we report that the AA content of dried tomatoes and fruit crisps is high, which is far more than their respective raw states. While many fruits are eaten raw, most vegetables are eaten cooked. It is well established that the cooking method alters the AA content of vegetables, and free AAs are sensitive to cooking methods [38,39]. Ito et al. [38] used different methods to cook vegetables, i.e., boiling, roasting in an oven, and a microwave. The total free AA content of vegetables decreased after boiling, with only 30 to 80% of the AA content remaining intact compared with the raw state. The “lost” AAs were recovered from the cooking liquid. Roasting in the oven caused an increase in the content of specific AAs due to the degradation of proteins and peptides into free AAs under heat treatment. Microwave cooking was reported only for cabbage, but resulted in around 80% retainment of free AAs, but interestingly, the amount of Tyr significantly increased. In our study, many vegetables were “boiled until tender” prior to analysis, which may have lowered the AA content.

Furthermore, the pattern of free AAs and the composition of the protein itself vary in plants even with the same type of structure at different stages of development, e.g., between young and old leaves [12]. The degree of ripeness/maturation at the time of sampling influences the protein content. Some fruits such as guava, mango and pineapple have enzymes that are structurally composed of proteins. As the fruits ripen, the protein content of the fruits increases up to the full-ripe stage and declines at the over-ripe stage due to the breakdown of proteins during senescence [40,41]. The origin of the food, genetic variability, and environmental conditions including climate, soil, time of harvest or post-harvest conditions (e.g., storage, processing) all affect the protein and AA content data of plant foods [35,42]. There are also reports comparing conventionally grown and organically produced crops. In two systematic reviews, the concentrations of proteins, AAs and nitrogen were found to be lower by 7–10% in organic crops [43,44]. Although the overall nutritional significance of slightly lower protein or AAs in organic plants is likely to be low, the difference may be important for low-AA/protein diets.

Only a limited number of fruits and vegetables were analysed due to the high costs of analysis. We also did not analyse a range of varieties and species and plants grown under different agronomic conditions. Unfortunately, most fruits and vegetables were only analysed once, and there was limited replicate analysis conducted, so information about the reliability and reproducibility of these results is unavailable. However, ion-exchange chromatography with post-column ninhydrin derivatization was used for the AA analysis. This method minimises interference from the sample matrix, and it is considered a reliable method of AA analysis.

The main cooking method, “boiled until tender”, used in this study, was likely to have lowered the AA content. Generally, there is limited information about the impact of different cooking methods and duration of cooking on the amino acid content of foods. It is probable that contemporary methods of cooking, such as microwaving or air frying, may lead to fewer losses of amino acid content. In future research, the effects of different common cooking methods on the amino acid content of plant foods should be compared. Methods should include air frying, vacuum frying, microwave-assisted or their combinations [45].

## 5. Conclusions

This study reported data on the AA content of several plant foods that are suitable for patients treated with a low-AA/protein diet, including many novel plant options. The inclusion of new and/or different plant foods is likely to help improve treatment management by increasing dietary variety and satiety. Accurate data on AA content will also enable dietitians to accurately calculate their dietary contribution and safely incorporate these plant foods into dietary plans for patients. The extension of amino acid analysis to an increased number of plant foods prepared by different cooking methods with replicate samples is necessary. Thus, future studies should be conducted to obtain more analytical data on the amino acid composition of plant foods. This would allow the incorporation of information on the amino acid content of a wider range of foods into national food databases and would provide an important resource for health professionals and patients with inherited amino acid disorders.

## Figures and Tables

**Table 1:** Amino acid contents of foods analysed by the National Society for Phenylketonuria (NSPKU).

Food Item	Food Category	Brand	Protein		Amino Acids (mg/100 g)				
			(g/100 g)	[Reference] Source for Protein Content ^a^	PHE	LEU	MET	TYR	LYS
Dragon fruit, raw	Fruits	-	1.2	[22] Weetch 2006	36	53	19	28	36
Kiwi fruit, raw	Fruits	-	0.8	[28] UK Food Database	35	54	18	22	43
Mulberries, raw	Fruits	-	1.4	[22] Weetch 2006	47	80	18	34	64
Passion fruit, raw	Fruits	-	2.6	[22] Weetch 2006	122	120	38	39	82
Apple crisps	Fruits	Marks & Spencer	1.8	Manufacturer data on website	27	44	8	12	45
	Fruits	Emilys	0.8		38	65	11	19	41
Melon crisps	Fruits	Nim’s	3.0		107	170	45	42	93
Pineapple crisps	Fruits	Nim’s	0.8		130	176	47	79	145
Avocado, raw ^b^	Fruits	-	1.9	[22] Weetch 2006	50	87	19	34	78
	Fruits	-	1.9	[22] Weetch 2006	46	73	20	33	65
	Fruits	-	1.9	[22] Weetch 2006	34	57	14	19	51
	Fruits	-	1.9	[22] Weetch 2006	49	80	19	33	65
Ackee, tinned	Vegetables	Dunn’s River	2.9	[28] UK Food Database	91	129	31	57	150
Baby corn, cooked	Vegetables	-	2.0	[22] Weetch 2006	78	128	36	71	119
Baby corn, tinned, cooked	Vegetables	-	2.0	[22] Weetch 2006	61	101	30	52	97
Baby spinach, raw	Vegetables	-	2.6	[28] UK Food Database	132	181	28	72	142
Banana blossom, tinned	Vegetables	-	1.3	[29] USDA Database	52	84	20	44	71
Breadfruit, tinned	Vegetables	Tropical Sun	1.3	[28] UK Food Database	27	40	8	17	35
Broccoli (purple sprouting), cooked	Vegetables	-	2.1	[28] UK Food Database	104	150	42	69	156
Broccoli (tenderstem), cooked	Vegetables	-	3.3	[28] UK Food Database	129	186	48	80	188
Butternut squash, cooked	Vegetables	-	0.9	[28] UK Food Database	29	40	12	20	39
Callaloo, tinned	Vegetables	Dunn’s River	1.8	[28] UK Food Database	96	142	32	54	97
Cassava, raw	Vegetables	-	0.6	[28] UK Food Database	10	10	0	10	20
Chard, yellow/rainbow, raw	Vegetables	-	1.8	Manufacturer data on website	52	75	19	37	72
Chard (ruby), raw	Vegetables	-	1.9	[29] USDA Database	144	221	53	107	198
Chard (Swiss), white, cooked	Vegetables	-	1.9	[29] USDA Database	152	220	52	117	118
Chestnuts, sweet, tinned	Vegetables	-	3.1	[22] Weetch 2006	137	222	36	72	172
Chayote, raw	Vegetables	-	0.8	[22] Weetch 2006	18	29	5	16	28
Choi Sum, raw	Vegetables	-	3.0	[22] Weetch 2006	139	195	31	77	159
Dudhi, cooked	Vegetables	-	1.8	[22] Weetch 2006	17	27	5	12	27
Dwarf beans, raw	Vegetables	Sainsbury’s	1.6	[28] UK Food Database	59	87	18	41	78
Eddoes, raw	Vegetables	-	1.5	[22] Weetch 2006	71	126	19	58	75
Hearts of palm, tinned, cooked	Vegetables	-	2.4	[22] Weetch 2006	83	155	43	63	160
Jackfruit, tinned	Vegetables	Biona Organic	1.3	[28] UK Food Database	29	46	8	19	46
Kale curly, cooked	Vegetables	-	3.4	[22] Weetch 2006	150	213	47	92	167
Kalettes, cooked	Vegetables	-	3.5	[22] Weetch 2006	127	215	58	94	228
Lotus roots, frozen, cooked	Vegetables	-	1.9	[22] Weetch 2006	63	72	30	56	72
Mangetout, cooked	Vegetables	-	3.6	[22] Weetch 2006	93	144	34	59	163
Microgreens, raw	Vegetables	-	2.0	[30] Kowitcharoen et al. 2021	99	130	8	17	110
Okra, cooked	Vegetables	-	2.8	[22] Weetch 2006	64	96	26	38	88
Pea shoots, raw	Vegetables	-	3.2	[22] Weetch 2006	274	324	86	156	355
Purple potato, cooked	Vegetables	-	3.6	[22] Weetch 2006	143	193	25	61	155
Rocket, raw	Vegetables	-	3.6	[22] Weetch 2006	143	193	25	61	155
Romanesco, cooked	Vegetables	-	3.0	[22] Weetch 2006	149	214	58	94	208
Runner beans, cooked	Vegetables	Sainsbury’s	1.6	[22] Weetch 2006	61	93	17	43	82
Samphire, cooked	Vegetables	-	1.2	[22] Weetch 2006	51	71	15	32	58
Stringless beans, raw	Vegetables	Sainsbury’s	1.6	[28] UK Food Database	44	70	16	36	63
Savoy cabbage, cooked	Vegetables	-	1.5	[22] Weetch 2006	52	85	22	43	89
Sugar snap peas, raw	Vegetables	Sainsbury’s	3.4	[22] Weetch 2006	75	100	21	47	107
Sugar snap peas, cooked	Vegetables	-	3.4	[22] Weetch 2006	88	139	24	55	142
Tomatoes, sunblush	Vegetables	Tesco	2.3	[28] UK Food Database	62	60	15	32	66
Tomatoes, sundried	Vegetables	Waitrose	4.8	Manufacturer data on website	95	123	35	93	126
Tomatoes, sundried	Vegetables	Baresa	3.0		75	95	23	45	80
Tomatoes, sundried	Vegetables	Marks & Spencer	5.4		152	171	46	90	164
Tomatoes, sundried	Vegetables	Morrisons	2.4		114	140	38	76	128
Tomatoes (cherry), slow roasted	Vegetables	Waitrose	2.7		95	123	35	93	126
Tomatoes, slow roasted	Vegetables	Tesco	2.3		62	60	15	32	66
Sweet potato, cooked	Vegetables	-	1.6	[28] UK Food Database	39	37	11	15	33
Sweet potato fries (frozen), cooked ^c^	Vegetables	Strong Roots	2.5	Manufacturer data on website	99	113	32	50	68
Sweet potato fries (frozen), cooked ^c^	Vegetables	McCain	2.3		92	101	28	41	61
Sweet potato fries (frozen), cooked ^c^	Vegetables	Waitrose	2.4		93	105	25	47	55
Vine leaves, cooked	Vegetables	-	3.0	[22] Weetch 2006	178	272	63	119	199
Watercress, raw	Vegetables	-	3.0	[22] Weetch 2006	26	31	6	17	26
Acai berry powder (organic)	Other plant foods	Green Origins	8.3	Manufacturer data on website	337	481	78	167	373
Aquafaba, (Chickpea Water)	Other plant foods	-	1.0		53	64	10	20	109
Flour, green banana	Other plant foods	Nihkan	5.0		126	176	36	53	99
Flour, cassava	Other plant foods	Tiana	1.0		50	75	18	23	75
Flour, potato	Other plant foods	-	0.1		1.1	2.2	0.3	0.7	1.8
Konnyaku/Konjac/Konjak	Other plant foods	-	0.0	[29] USDA Database	0.7	1.0	0.0	ND	0.2
Mung bean vermicelli	Other plant foods	Triple Win Ltd.	0.5	Manufacturer data on website	4	7	1	1	6
Rice noodles	Other plant foods	Amoy	1.8		75	115	48	52	50
Rice noodles	Other plant foods	Tiger Tiger	3.0		105	156	46	65	70
Tamarind paste	Other plant foods	Waitrose	2.4		160	210	52	103	169

Abbreviations: Phe, phenylalanine; Leu, leucine; Met, methionine; Tyr, tyrosine; Lys, lysine; ND, not determined. Many of the vegetables were cooked (boiled or steamed until tender) to represent the condition of the food at the time of serving); they were drained and blended to pulp prior to sampling. ^a^ Protein values were obtained from different sources/references [22,28,29,30], [-] Manufacturer data reported on grocery websites. ^b^ Avocado was purchased from different supermarkets. ^c^ All three manufactured sweet potatoes were lightly coated with a gluten-free flour (e.g., rice flour, amaranth flour or corn flour). Hence, the protein and amino acid values might be slightly different than uncoated, cooked sweet potatoes.

**Table 2:** The amount of protein (g) and amino acids (mg) per 100 g of fruits and vegetables analysed by NSPKU.

	Protein (g/100 g)	Phe (mg/100 g)	Leu (mg/100 g)	Met (mg/100 g)	Tyr (mg/100 g)	Lys (mg/100 g)
Fruits (*n* = 8) ^a^						
Mean ± SD	1.7 ± 0.6	52.4 ± 28.9	75.5 ± 22.3	20.6 ± 7.2	30.3 ± 6.8	60.5 ± 26.1
Median [range]	1.9 [0.8–2.6]	47 [34–122]	77 [53–120]	19 [14–38]	33 [19–39]	65 [36–82]
Vegetables (*n* = 48) ^b^						
Mean ± SD	2.4 ± 1.0	89.3 ± 51.0	126.2 ± 70.1	28.6 ± 17.6	57.5 ± 31.9	114.6 ± 64.7
Median [range]	2.3 [0.6–5.4]	81 [10–274]	125 [10–324]	26 [0–86]	56 [10–156]	109 [20–355]
Fruits and Vegetables Total (*n* = 56)						
Mean ± SD	2.3 ± 1.0	84.0 ± 50.0	119.0 ± 67.8	27.5 ± 16.7	53.6 ± 31.2	106.9 ± 63.0
Median [range]	2.0 [0.6–5.4]	73 [10–274]	101 [10–324]	24 [0–86]	46 [10–156]	89 [20–355]

Abbreviations: Phe, phenylalanine; Leu, leucine; Met, methionine; Tyr, tyrosine; Lys, lysine; SD, standard deviation; NSPKU, National Society for Phenylketonuria. ^a^ Fruit crisps were not included in the analysis. ^b^ Manufactured sweet potato fries (cooked) were not included in the analysis due to the extra protein content from flour (e.g., rice flour, corn flour, etc.) used as a coating.

**Table 3:** The percentage (%) and the amount (mg) of amino acids per gram of protein in fruits and vegetables.

	Percent of Amino Acids per Gram of Protein					Amino Acid Content (mg) per Gram of Protein				
	% Phe	% Leu	% Met	% Tyr	% Lys	Phe (mg)	Leu (mg)	Met (mg)	Tyr (mg)	Lys (mg)
Fruits (*n* = 8) ^a^										
Mean ± SD	3.1 ± 1.0	4.6 ± 1.1	1.3 ± 0.5	1.9 ± 0.6	3.7 ± 0.9	31 ± 10	46 ± 11	13 ± 5	19 ± 6	37 ± 9
Median [range]	3 [2–5]	5 [3–7]	1 [1,2]	2 [1–3]	3 [3–5]	28 [18–47]	45 [30–68]	12 [7–23]	18 [10–28]	34 [27–54]
Vegetables (*n* = 48) ^b^										
Mean ± SD	3.7 ± 1.6	5.3 ± 2.4	1.2 ± 0.6	2.4 ± 1.2	4.8 ± 1.9	37 ± 16	53 ± 24	12 ± 6	24 ± 12	48 ± 19
Median [range]	4 [1–9]	5 [1–12]	1 [0–3]	2 [1–6]	5 [1–11]	35 [9–86]	54 [10–116]	11 [0–28]	24 [6–62]	48 [9–111]
Fruits and Vegetables Total (*n* = 56)										
Mean ± SD	3.6 ± 1.5	5.2 ± 2.2	1.2 ± 0.6	2.4 ± 1.1	4.6 ± 1.8	36 ± 15	52 ± 23	12 ± 6	24 ± 11	46 ± 18
Median [range]	3 [1–9]	5 [1–12]	1 [0–3]	2 [1–6]	5 [1–11]	34 [9–86]	48 [10–116]	11 [0–28]	23 [6–62]	46 [9–111]

Abbreviations: Phe, phenylalanine; Leu, leucine; Met, methionine; Tyr, tyrosine; Lys, lysine; SD, standard deviation. ^a^ Fruit crisps were not included in the analysis. ^b^ Manufactured sweet potato fries (cooked) were not included in the analysis due to the extra protein content from flour (e.g., rice flour, corn flour, etc.) used as a coating.

## Data Availability

Data are contained within the article.

## Supplementary Materials

The following supporting information can be downloaded at: https://www.mdpi.com/article/10.3390/nu15102387/s1, Table S1: Correlation between protein (grams) and amino acids (mg) per 100 g food for fruits and vegetables analyzed by NSPKU.
