# Supplementary material for: Amino Acid Analyses of Plant Foods Used in the Dietary Management of Inherited Amino Acid Disorders
Source: Nutrients. 2023 May 19;15(10):2387. doi: 10.3390/nu15102387 (PMC10222841; doi:10.3390/nu15102387)
Supplement: Supplementary file 1 [file nutrients-15-02387-s001.zip › nutrients-2393575-supplementary.pdf]

Supplementary Table S1. Correlation between protein (grams) and amino acids (mg) per 100 g food for fruits and vegetables analyzed by NSPKU.

Variables	Amino acids in fruits (*n* = 8)[a]						Amino acids in vegetables (*n* = 48)[b]					
	Protein	Phe	Leu	Met	Tyr	Lys	Protein	Phe	Leu	Met	Tyr	Lys
**1. Protein**	-						-					
**2. Phenylalanine**	0.74	-					0.61	-				
**3. Leucine**	0.80	0.92	-				0.57	0.97	-			
**4. Methionine**	0.63	0.98	0.84	-			0.52	0.88	0.89	-		
**5. Tyrosine**	0.57	0.69	0.85	0.67	-		0.55	0.90	0.91	0.95	-	
**6. Lysine**	0.80	0.70	0.91	0.58	0.78	-	0.61	0.93	0.94	0.91	0.88	-

Abbreviations; Phe, phenylalanine; Leu, leucine; Met, methionine; Tyr, tyrosine; Lys, lysine; NSPKU, National Society for Phenylketonuria.

[a] Fruit crisps were not included in the analysis.

[b] Branded sweet potato fries (cooked) were not included in the analysis due the extra protein content from flour (e.g. rice flour, corn flour, etc.) used as a coating.
